# Rampant centrosome amplification underlies more aggressive disease course of triple negative breast cancers

**DOI:** 10.18632/oncotarget.3402

**Published:** 2015-03-19

**Authors:** Vaishali Pannu, Karuna Mittal, Guilherme Cantuaria, Michelle D. Reid, Xiaoxian Li, Shashikiran Donthamsetty, Michelle McBride, Sergey Klimov, Remus Osan, Meenakshi V. Gupta, Padmashree C.G. Rida, Ritu Aneja

**Affiliations:** ^1^ Department of Biology, Georgia State University, Atlanta, GA 30303, USA; ^2^ Department of Gynecologic Oncology, Northside Hospital Cancer Institute, Atlanta, GA 30342, USA; ^3^ Department of Pathology, Emory University Hospital, Atlanta, GA 30322, USA; ^4^ Department of Mathematics and Statistics, Georgia State University, Atlanta, GA 30303, USA; ^5^ Neuroscience Institute, Georgia State University, Atlanta, GA 30303, USA; ^6^ Clinical Pathology & Anatomic Pathology, West Georgia Hospitals, LaGrange, GA 30240, USA; ^7^ Institute of Biomedical Sciences, Georgia State University, Atlanta, GA 30303, USA

**Keywords:** centrosome amplification, triple negative breast cancer, metastasis, disease prognosis

## Abstract

Centrosome amplification (CA), a cell-biological trait, characterizes pre-neoplastic and pre-invasive lesions and is associated with tumor aggressiveness. Recent studies suggest that CA leads to malignant transformation and promotes invasion in mammary epithelial cells. Triple negative breast cancer (TNBC), a histologically-aggressive subtype shows high recurrence, metastases, and mortality rates. Since TNBC and non-TNBC follow variable kinetics of metastatic progression, they constitute a novel test bed to explore if severity and nature of CA can distinguish them apart. We quantitatively assessed structural and numerical centrosomal aberrations for each patient sample in a large-cohort of grade-matched TNBC (*n* = 30) and non-TNBC (*n* = 98) cases employing multi-color confocal imaging. Our data establish differences in incidence and severity of CA between TNBC and non-TNBC cell lines and clinical specimens. We found strong correlation between CA and aggressiveness markers associated with metastasis in 20 pairs of grade-matched TNBC and non-TNBC specimens (*p* < 0.02). Time-lapse imaging of MDA-MB-231 cells harboring amplified centrosomes demonstrated enhanced migratory ability. Our study bridges a vital knowledge gap by pinpointing that CA underlies breast cancer aggressiveness. This previously unrecognized organellar inequality at the centrosome level may allow early-risk prediction and explain higher tumor aggressiveness and mortality rates in TNBC patients.

## INTRODUCTION

As the name foretells, triple-negative breast cancers (TNBC) do not over-express the estrogen, progesterone, or Her2 receptors, thus precluding patient response to several targeted therapies available in the clinic. More so, TNBC preferentially afflicts women of African descent and is characterized by high aggressiveness, recurrence, metastases and mortality rates [1–3]. About 40% of all breast cancer cases in pre-menopausal AA patients are TN (5-year survival of ~80% as opposed to ~90% in European American patients), putting this patient subgroup at a higher risk for poorer outcomes [4].

Recent literature points out an intriguing correlation between BRCA1 (breast cancer suppressor gene 1) mutation carriers and TNBC status. About 70% of women with breast cancer carrying a BRCA1 mutation belong to the triple-negative subtype [5]. Beyond a crucial role of BRCA1 in regulating centrosome duplication, BRCA1 is also known to interact with a variety of proteins that regulate centrosome duplication, including BRCA2 (breast cancer suppressor gene 2), CDK2/Cyclin A, CDK2/Cyclin E, Gadd45, p21, p53 and Rb [6–8]. Furthermore, targeted disruption of BRCA1 results in centrosome amplification (CA), confirming that BRCA1 serves as a negative regulator for centrosome duplication [9]. These data are consistent with the notion that BRCA1 and BRCA2 tumor suppressor genes directly maintain genomic stability, and affirm a causative link between BRCA1 mutations and centrosomal overload that is associated with extensive chromosomal instability (CIN) found in TNBC patients. Therefore, amplified centrosomes may present a fascinating, readily quantifiable prognostic marker and druggable target for TNBCs, currently plagued by insufficiency of molecular targets that precludes their treatment and optimal management.

Centrosomal abnormalities and CIN strongly characterize pre-invasive *in situ* ductal carcinomas, thus incriminating these anomalies in fueling tumor progression and metastases. Thus far, a thorough quantitative comparison of centrosomal aberrations in breast tumor subtypes with inherently different metastatic capability has never been reported. Herein, we performed a comprehensive quantitative analysis of centrosomal abnormalities in breast tumors to establish differences in incidence and severity of CA (structural and numeral) between grade-matched TNBC (*n* = 30) and non-TNBC (*n* = 98) patients. Intriguingly, we found significant correlation of CA status with patient outcomes wherein we ascertained that patients exhibiting higher centrosome aberrations (> 20%) had lower Progression free survival (PFS) than patients with lower centrosome aberrations (< 20%). We also established a strong association between CA markers and markers of breast tumor aggressiveness, suggesting that robust CA underlies acquisition of aggressive phenotypes.

Our results generate compelling foregrounds to establish CA as a quantifiable property of low-grade tumors that can predict the risk of a tumor being or becoming an aggressive one. A validated method to quantify this cell-biological cancer-specific organellar trait can provide clinicians with a method to stratify low-grade tumors into high- and low-risk subgroups and may enable channeling of patients into optimal treatment paths to reduce existing disparities in breast cancer patient outcomes.

## RESULTS

### *In silico* overexpression of CA-associated genes is correlated with reduced survival and triple-negative subtype

**Figure 1:**
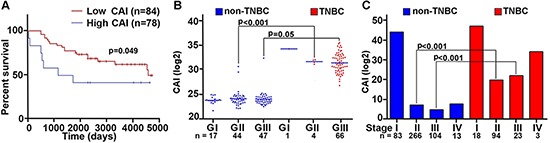
Breast tumors with higher CAI have lower survival rate compared to low CAI tumors **(A)** Overall survival plots for high and low CAI groups. High CAI patients had lower overall survival (*n* = 84) compared to low CAI patients (*n* = 78) in *in silico* data. **(B)** Scatter-plot comparing the CAI in TNBC and grade-matched non-TNBC patients (*p* < 0.001 for Grade II TNBC and non-TNBC patients). **(C)** Bar graphs comparing the CAI in TNBC and non-TNBC patients considering stages (*p* < 0.005 for stage-matched TNBC and non-TNBC patients). (Lower n number in the survival analysis is due to the limited availability of survival data for all patients in the referred databases.)

Previous studies in solid tumors have alluded to an association between centrosomal abnormalities and advanced disease, aneuploidy, and an aggressive clinical course. These studies however lacked rigorous quantitation of the centrosomal abnormalities and have not explored whether centrosomal abnormalities are accompanied by any changes in the expression patterns of centrosomal genes. Given that there are differences in aggressive behavior between TNBC and non-TNBC patients, we investigated whether these histologically-distinct breast cancer subtypes might differ in the expression levels of centrosomal genes. To this end, we mined publically-available microarray data of breast cancer patients to evaluate gene expression levels for major structural centrosomal proteins, both centriolar (centrin) and pericentriolar (pericentrin and γ-tubulin). To gain deeper insights into centrosomal aberrations, we included genes whose dysregulation is implicated in CA (polo-like kinase 4 and cyclin E). We calculated a cumulative gene expression-based centrosome amplification index (CAI) by adding log transformed, normalized gene expression for CETN2 (centrin-2), TUBG1 (γ-tubulin), PCNT2 (pericentrin), PLK4 (polo-like kinase 4) and CCNE1 (cyclin E) genes. Given that cancer is a clonally evolving disease and CA could arise due to dysregulation of different genes in different cancers and even distinct cancer cell clones, we chose to select a panel of five centrosomal genes instead of a single gene. First, we evaluated the relationship of higher CA, as assessed by CAI, with disease aggressiveness, as determined by overall survival (OS). OS was calculated as the number of days from diagnosis to death or last follow-up if death was not recorded. Irrespective of receptor status (TNBC *n* = 101, non-TNBC *n* = 61), patients with higher CAI (*n* = 78) had lower OS (*p* = 0.049) than patients with lower CAI (*n* = 84) (Fig. 1A). Intriguingly, high CAI group was composed of ~60% TNBC cases whereas the low CAI group composed of ~38% TNBC cases, thus indicating that TNBCs tend to have higher CAI as compared to non-TNBCs. Further analysis of another dataset of 138 TNBC and 466 non-TNBC samples clearly showed significantly higher CAI in TNBCs compared to non-TNBCs, even when they were (a) grade-matched (Fig. 1B), or (b) stage-matched (Fig. 1C).

In sum, these data suggest that more aggressive disease course in TNBC is accompanied by significant overexpression of centrosomal genes. Additionally, overexpression of centrosomal genes is strongly associated with poorer OS.

### TNBC patients have higher centrosome amplification than non-TNBC

**Figure 2:**
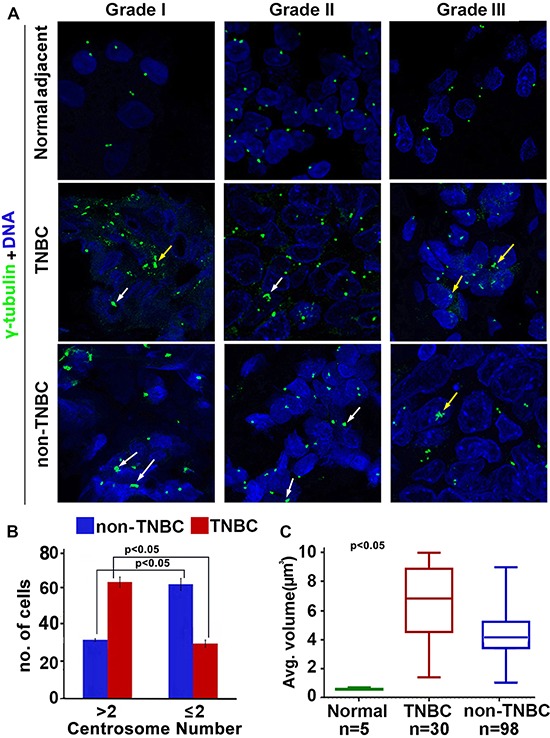
Comparison of extent of centrosome amplification in grade-matched TNBC and non-TNBC patients **(A)** Representative confocal micrographs depicting the status of centrosome amplification in histological Grade I, Grade II and Grade III TNBC and non-TNBC tissues. Centrosomes were labelled with γ-tubulin antibody (green) and DNA was stained with DAPI (blue) Yellow arrows indicate numerical amplification and white arrows indicate structural amplification. Scale bar 5 μm. **(B)** Bar graph representation of number of cells out of 500 showing supernumerary centrosomes (> 2 or ≤ 2) in TNBC and non-TNBC tissue samples. **(C)** Box whisker graph representation of average volume of centrosomes in normal, TNBC and non-TNBC tissue samples. At least 500 cells were counted in each case (ANOVA, *p* < 0.05).

Our *in silico* data analysis yielded significantly elevated CAI in TNBC women compared to non-TNBC. Thus, we visualized amplified centrosomes in grade-matched tissues from TNBC (*n* = 59) and non-TNBC (*n* = 116) patients (Fig. 2A) employing multicolor confocal immunofluorescence microscopy. Centrosomes were labelled by γ-tubulin (green) antibody, wherein centrosomal aberrations were determined by abnormal number of γ-tubulin spots (more than two) as well as by increased volume over normal centrosomal volume in breast epithelial cells from normal adjacent tissues. Fig. 2A shows representative confocal immunomicrographs depicting centrosomes (γ-tubulin, green) and nuclei (DAPI, blue) from normal breast tissue and breast cancer tissue from grade-matched (Grade II) TNBC and non-TNBC patients. Cells with more than two centrosomes were estimated by examining and counting centrosomes in atleast 500 cells/slide. Fig. 2B shows that the number of cells harboring extra centrosomes were twice as much higher in TNBC samples (62, *n* = 30) versus non-TNBC (30, *n* = 98) (*p* < 0.05) in a grade-matched background. We next measured centrosomal volumes in all specimens using the three-dimensional measurement module from the Zeiss imaging software. While mean centrosomal volume in normal breast epithelial cells was 0.22 μm^3^ (ranging from 0.08 to 0.76 μm^3^), the mean volume of γ-tubulin-stained spots analyzed in at least 500 tumor cells from each patient sample was 4.85 μm^3^, which is ~15 times higher than the centrosomal volume in normal cells. Fig. 2C shows that centrosome volume was significantly higher in TNBC samples (*n* = 30) (average = 6.8 μm^3^) compared to grade-matched (Grade II) non-TNBC (*n* = 98) samples (average = 4.2 μm^3^) (*p* < 0.05). Additionally, we calculated the total centrosome amplification as a percentage by adding percent cells harboring more than two centrosomes and percent cells harboring centrosomes with volume larger than 0.76 μm^3^. Our analysis revealed that on an average, ~68% of cells in TNBC samples exhibited centrosome amplification as compared to ~45% in non-TNBC samples (*p* < 0.05).

**Figure 3:**
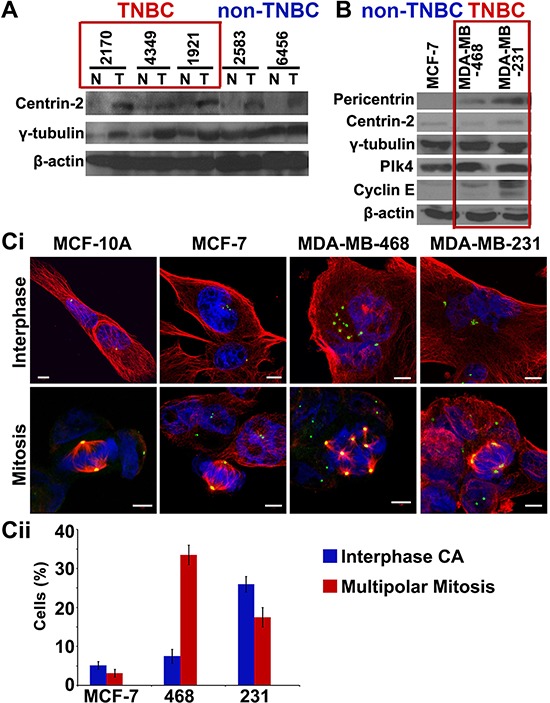
TNBC tumors and cell lines show higher expression of centrosomal markers **(A)** Immunoblots for 5 paired breast tumor (T) and normal adjacent (N) tissues from grade-matched TNBC and non-TNBC patients showing expression levels of centrosomal markers. **(B)** Immunoblots of centrosomal markers in MCF-7 (non-TNBC), MDA-MB-468 and MDA-MB-231 (TNBC) cell lines. **(Ci)** Immunofluorescence micrographs showing MCF-10A cells (near-normal), MCF-7 cells (non-TNBC) and MDA-MB-468 and MDA-MB-231 cells (TNBC) in interphase and mitotic state, stained for γ-tubulin (green), α-tubulin (red) and DAPI (blue). **(Cii)** Bar graph quantitation of percent cells depicting multipolar mitosis and centrosomal amplification in interphase in MCF-7 (non-TNBC) and MDA-MB-468 and MDA-MB-231 (TNBC) cell lines. (Only Grade II TNBC and non-TNBC samples were matched because of limited n numbers for Grade I TNBC and Grade III non-TNBC samples in our dataset.)

We next compared the expression levels of centrosomal proteins (centrin-2 and γ-tubulin) in fresh-frozen tumors and uninvolved adjacent tissue from 20 pairs of grade-matched TNBC and non-TNBC patients. Immunoblotting of tissue lysates showed higher expression of two centrosomal proteins (centrin-2 and γ-tubulin) in TNBC than in non-TNBC patients (Fig. 3A). In addition, breast cancer cell lines derived from TNBC patients (MDA-MB-468, MDA-MB-231) showed (a) higher incidence and severity of centrosome amplification (Fig. 3Ci, ii) (b) elevated expression of centrosomal (pericentrin, centrin-2, γ-tubulin) and centrosome amplification markers (Plk4 and cyclin E), compared to non-TNBC-derived (MCF-7) lines (Fig. 3B). Intriguingly, the extent of amplification was considerably lower in cell lines (5–30%) compared to patient tissue samples (15–80%), irrespective of the receptor status. This observation highlights a previously unrecognized discordance between human tumor tissues and established cell lines. This also underscores the limitations of *in vitro* cell lines as a model system for establishing and testing centrosome-targeted therapies.

### Centrosome amplification status correlates strongly with metastatic disease and progression-free survival in patient samples

**Figure 4:**
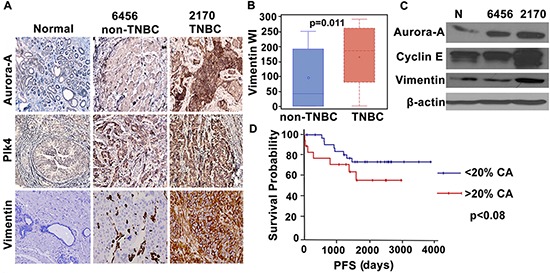
Breast tumors in TNBC patients show higher expression of centrosomal proteins and aggressiveness markers **(A)** Micrographs showing immunohistchemical staining for Aurora-A, Plk4 (centrosomal amplification markers) and vimentin (breast cancer metastasis marker) in normal and cancer tissue from representative grade-matched TNBC (2170) and non-TNBC (6456) patients. **(B)** Box whisker graph showing significantly higher expression of vimentin in TNBC samples when compared to grade-matched non-TNBC samples. **(C)** Immunoblots for normal, TNBC (2170) and non-TNBC (6456) tissue samples showing Aurora-A, Cyclin E and vimentin expression. **(D)** Progression free survival (PFS) plot for the patients with higher (> 20%) CA (red) and lower (< 20%) CA (blue). (Only Grade II TNBC and non-TNBC samples were matched because of limited n numbers for Grade I TNBC and Grade III non-TNBC samples in our dataset.)

We next evaluated expression levels of molecules implicated in CA by immunohistochemical staining of paraffin-embedded tumor and uninvolved adjacent tissue in 20 pairs of grade-matched (Grade II) TNBC and non-TNBC samples. Immunohistochemical data strongly suggested overexpression of well-established centrosome amplification markers (Aurora-A and Plk4) in TNBC compared to non-TNBC samples (Fig. 4A). The same samples were also immunostained for vimentin, a breast cancer aggressiveness marker. As expected, TNBC samples exhibited significantly higher expression of vimentin (Fig. 4B). Representative images of two sets of grade-matched breast tumor samples (one TNBC, one non-TNBC) exhibiting different expression levels of centrosomal proteins, show that TNBC samples exhibited higher expression levels of these proteins than non-TNBC samples and normal tissue (Fig. 4A). Statistical analysis of 20 pairs of grade-matched TNBC and non-TNBC samples revealed a strong positive correlation between Plk4 and vimentin (*r* = 0.58, *p* < 0.02). Immunoblots shown in Fig. 4C also support our observation that centrosomal markers (Aurora-A, Plk4 and cyclin E) exhibit similar expression patterns and trends as metastasis markers (vimentin) in breast tumor samples. Furthermore, we evaluated the relationship of higher centrosome aberrations (as assessed by %CA in Fig. 2Bi) with progression-free survival (PFS) in breast cancer patients. PFS was calculated as the number of days from diagnosis to the first local recurrence or metastasis (if one occurred), or the last follow-up if the patient did not progress. Irrespective of receptor status (*n* = 120), patients with higher centrosome aberrations (> 20%) had lower PFS (*p* < 0.08) than patients with lower centrosome aberrations (< 20%) (Fig. 4D). Interestingly, a majority (~72%) of high CA (high-risk) group were TNBC while low CA (low-risk) group were largely non-TNBC (~60%).

### Cells with supernumerary centrosomes show higher migration velocity and net displacement compared to cells with normonumerary centrosomes

**Figure 5:**
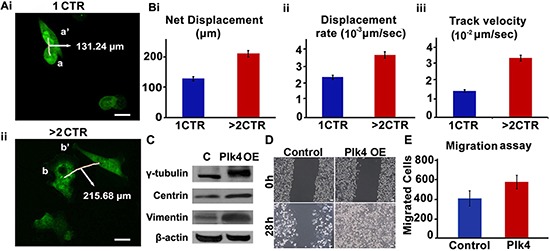
Cells with amplified centrosomes show higher migration **(Ai, ii)** Time lapse images over 18 h of GFP-centrin-MDA-MB-231 cells with one (i-top panel) versus four (ii-bottom panel) centrosomes. Trajectories of 10 cells each were captured over 18 h (6 frames/h). Data was analyzed using Volocity 3.0 software and average net displacement, displacement rate and velocity measurements were generated for the respective cells with respect to cell centroids. Scale bar 10 μm. Quantitation of net displacement **(Bi)** displacement rate **(Bii)** track velocity **(Biii)** for cells with 1 and > 2 centrosomes are shown in bar graphs. Track velocity is the rate at which the cell traces the entire path between point a and a'. Displacement rate is the rate at which cell covers the distance from point a to a'. **(C)** Immunoblots showing protein expression levels of γ-tubulin, centrin and vimentin in MCF-10A cells expressing either inducible truncated Plk4 (denoted as C) or inducible wild-type Plk4 (denoted as Plk4 OE). **(D)** Brightfield microscopic images showing wound healing capacity of MCF-10A cells expressing either inducible truncated Plk4 (denoted as Control) or inducible wild-type Plk4 (denoted as Plk4 OE) at 0 h and 28 h. Scale bar 10 μm. **(E)** Bar graph showing the number of invaded cells in Boyden chamber assay performed with control (truncated Plk4) and wild-type Plk4 overexpressing MCF-10A cells. CTR = centrosomes.

We next asked if extra centrosomes were associated with enhanced cell migration. To discern this, we performed a cell migration assay using time-lapse imaging wherein migration of MDA-MB-231 cells (stably transfected with GFP-tagged centriolar protein, centrin) harboring 1 centrosome or > 2 centrosomes was observed. Fig. 5A shows representative data for a pair of cells observed upon 18 h time-lapse imaging, where a cell with 1 centrosome migrated from a to a' (Fig. 5Ai) and a cell with 4 centrosomes moved from b to b' (Fig. 5Aii). Quantitation of merged time-lapse sequences (10 min apart, collected over 18 h) showed higher average net displacement (78%), displacement rate (55%) and track velocity (~2 fold enhancement) of cells with > 2 centrosomes compared to cells with 1 centrosome (Fig. 5Bi,ii,iii). These data strongly suggest that overabundance of centrosomes enhances migratory ability in cancer cells. Having established a strong correlation between extra centrosomes and cell migration in MDA-MB-231, an aggressive breast cancer cell line, we next asked if centrosome amplification directly and independently impacted the migration potential of a near-normal immortalized breast epithelial cell line, MCF-10A. We evaluated if generation of extra centrosomes by genetic means will impact migration and invasion kinetics in breast epithelial cells. To this end, we used MCF-10A cells engineered to facilitate inducible overexpression of wild-type Plk4 or truncated Plk4 (1-608, negative control), upon doxycycline treatment for 48 h. CA induced 48 h after Plk4 induction was confirmed by immunofluorescence staining for γ-tubulin (green) (data not shown) and change in γ-tubulin levels by immunoblotting in cells overexpressing wild-type Plk4 (denoted as Plk4 OE in Fig. 5C, whereas no amplification was observed in cells overexpressing truncated Plk4 (denoted as C in Fig. 5C). We also observed a significant increase in vimentin levels along with centrosomal proteins upon doxycycline-based induction of CA (Fig. 5C). We then performed a classical wound healing assay to assess the migratory capacity of cells with amplified centrosomes (~80% cells harboring extra centrosomes) as compared to the control cells. We found that cells with CA filled the scratch wound in less than half the time as control cells (28 h as compared to 60 h) (Fig. 5D). Additionally, an increase in the invasion capacity of wild-type Plk4 overexpressing cells was observed in a Boyden chamber assay. Fig. 5E shows that a significantly higher number of cells invaded the bottom chamber of the filter after 48 h of incubation. We also confirmed these observations by inducing centrosome amplification via pharmacological manipulation. We experimentally induced centrosome amplification in MDA-MB-231 cells by aphidicolin treatment (5 μg/ml for 48 h). Centrosome amplification upon treatment was confirmed by immunofluorescence staining for γ-tubulin (Suppl. Fig. 1A). An increase in the invasion capacity of treated cells was observed via Boyden chamber assay (Suppl. Fig. 1B).

These data collectively underscore the critical role of centrosomes in facilitating directed cell migration and invasion. These observations lay the experimental foundation that link CA, an important cellular feature of certain cancers, to more aggressive phenotypes and provide a tantalizing possibility that organellar-level differences may serve as a risk predictor of aggressiveness in breast cancer patients.

## DISCUSSION

A century ago, Theodore Boveri proposed that subtle mitotic errors in dividing cells are responsible for generating aneuploidy and chromosomal instability, and thus propelling tumorigenesis [10]. This pioneering theory originated from his observations that increased number of centrosomes lead to multipolar mitosis and highly aneuploid daughter cells. Since then, several landmark studies have demonstrated that the centrosome plays an important role in erection of a fusiform spindle apparatus that ensures high-fidelity chromosome segregation to produce two genetically-identical progeny cells [11, 12]. It is now well-established that abnormal number of centrosomes most often result in aberrant mitotic divisions and aneuploidy, all of which are frequently observed in many solid and hematological human cancers [13–18].

In addition, chromosomal instability underlies generation of aneuploidy over time, and has been associated with various prognosticators in breast cancer. All these studies point towards a strong correlation between centrosome amplification and tumor progression, recurrence and poor survival yet the mechanistic aspects of this relationship have remained elusive. Recently, Godinho et al. provided convincing cell-based evidence to prove that centrosome amplification can cause oncogene-like effects to promote cellular invasion in mammary epithelial cells [19]. These findings assert that cytoskeletal modifications at the structural level driven by centrosome amplification engenders transformation potential in normal epithelial cells and is directly responsible for tumor initiation and progression. This study clearly substantiates our published hypothesis that cells endowed with extra centrosomes possess a cytoskeletal edge over other cells, thus imparting enhanced cell polarization, Golgi-mediated vesicular trafficking and invasion potential, leading to increased metastatic progression.

Indeed, a miscellany of centrosomal defects such as increase in number, size, and atypical structure comprise centrosome amplification. Centrosomal amplification in human cancers is of two kinds: structural and numerical [20, 21]. Structural defects can be due to abnormal centriole structure and/or abnormal amount of pericentriolar material (PCM). While detection of alterations in centriole size is precise using centriole-specific antibody, an increased amount of PCM, detected by a pericentriolar antibody, can present complex ambiguous scenarios. One possibility is increased PCM, making it a ‘true structural defect’. Another possibility is that supernumerary centrosomes that are tightly clustered during interphase may appear as a large centrosome, and thus in fact appear to be a ‘structural defect’. In several cases, one can notice supernumerary centrosomes with increased PCM, making it both a structural and numerical defect. These issues render quantitation of centrosomal defects very subjective and dependent on the viewer's discretion. Therefore, better methods to systematically assess and distinguish centrosome abnormalities are much needed. Numerical defects in the form of centrosome amplification have been widely described in human cancer, but not much is known about the structural defects. It is reasonable to speculate that both numerical and structural defects may distinctly shape the course of tumor progression. We reason that in low grade tumors, numerical defects that manifest as amplified centrosomes may advantageously serve cancer cells during an erroneous mitosis by offering them a means to generate an array of clones that form the basis of intratumor heterogeneity, the Holy Grail of cancer chemotherapy. Once these heterogeneous clones attain desirable karyotypes, we conjecture that individual centrosomes tend to cluster which may endow cells with cytoskeleton-derived mechanical advantages for directional migration. This might partly explain why certain non-invasive lesions transform into highly aggressive tumors with metastatic capabilities while others tend to remain indolent. This is in consonance with the fact that an increased centrosomal complement displays an augmented microtubule nucleation capacity [13]. We envision that a higher microtubular density in cancer cells in conjunction with the action of the actin cytoskeleton may provide cells with protrusion capability for faster migration through the ECM [22]. Centrosomes and the microtubules they nucleate together establish the nuclear-centrosomal axis, which defines direction of cell movement, and controls post-mitotic Golgi reassembly [23]. Essentially, microtubules nucleated on the juxta-centrosomal Golgi serve as tracks for Golgi-derived vesicles carrying proteins essential for leading edge protrusion, and matrix remodeling [22]. Our data in MDA-MB-231 cells (where 20–30% of cells display CA) showed that cells with extra centrosomes migrate ~2-fold faster than cells with normal centrosome number (Fig. 5A, 5B). In addition to facilitating faster cellular movement, it is likely that supernumerary centrosomes may impart cells with an enhanced stroma-penetrating capacity through an inventory of mechanisms including augmentation of microtubule-dependent MMP secretion.

Currently-used prognostic indicators in breast cancer (e.g. Ki67) do not predict metastases risk accurately in early- tumors. Expensive commercial multigene expression assays such as OncotypeDx provide a “Recurrence Score” and while treatment paths for patients with scores < 18 (low-risk) and > 31 (high-risk) are relatively unambiguous, 34% of patients with scores between 18–31 lack additional markers to guide optimal [24]. Given that breast cancers harbor amplified centrosomes, a quantitative estimation of centrosomal abnormalities in fine-needle aspirates or paraffin-embedded samples may prove to be a simple, sensitive, and easily quantifiable risk-predicting biomarker.

Precise prognosis suffers challenges owing to various confounding factors with bearing on breast cancer patient outcomes such as race, age, receptor-status, molecular signatures and so on. For instance, among African-American women with breast cancer, there is an estimated 20–40% chance of the breast cancer being triple-negative [4, 25]. It is well known that TNBC accounts for ~15% of all breast cancer in the United States. It seems to be more common in (a) young women, (b) women of African ancestry, and (c) individuals with BRCA1 mutations [26]. Cancer recurs in ~30% of TNBC patients at an early stage, typically within three to four years following treatment. Further studies are required to understand why premenopausal women and women in some ethnic groups have higher rates of triple-negative breast cancer than other groups of women, and what mechanisms underlie the aggressive traits of TNBCs. Such studies will be critical in order to device relevant treatment strategies specific for the particular group pf patients or tumor subtypes. However, finding suitable targeting pathways in TNBC is proving to be a challenge, particularly because these biologically aggressive tumors do not depend on one dominant pathway, unlike ER-positive or Her2-positive tumors. Several studies have led to the realization that TNBC tumors are a mixed bag and this extensive heterogeneity reported in TNBC provides a strong indication that CA that underlies chromosomal instability driven karyotypic diversity could be the underlying cause of the aggressive nature of this breast cancer subtype. Recent reports focused on identifying the key genetic and molecular features of individual TNBC tumors led to the identification of 7 TNBC subtypes, as follows: basal-like 1 (BL1); basal-like 2 (BL2); immunomodulatory (IM); mesenchymal (M); mesenchymal stem–like (MSL); luminal androgen receptor (LAR); and unstable (UNS) [27]. Such a classification based on differential gene and molecular expression is a significant effort in unmasking specific susceptibilities of tumors, which can be targeted for tailored therapies. While the enormous effort and cost associated with such gene expression based assays limit their feasibility and prompt use in the clinic, non-invasive centrosome-based detection methods (e.g. in fine-needle aspirate cytology) may allow early detection of fatal breast cancers and distinguish them from benign forms. Since majority of breast cancers are characterized by centrosome amplification, the extent, severity and type of amplification can provide insights into the metastatic propensity of certain tumors as opposed to others. In this study, we have clearly demonstrated a positive correlation between markers of centrosome amplification and vimentin expression in breast cancer patients. These data lay compelling grounds for the exploration of centrosomal defects as cell-biological traits of non-invasive lesions that can potentially determine metastatic risk and meaningfully contribute towards the dawn of precision medicine.

## MATERIALS AND METHODS

### *In silico* analysis of CAI

One channel microarray data were collected from Gene Expression Omnibus (GEO) database and Cancer Genome Atlas (TCGA) and processed using Robust Multiarray (RMA) normalization, and was further used for gene expression analysis. The lists of the GSE ID's are given in Supplementary table 1. Log2n transformed expression levels of Plk4, Aurora-A, Centrin-2, *γ*-tubulin and pericentrin genes were extracted from the TCGA and GEO patients and a summation was calculated to generate a CAI for TNBC and Non-TNBC patients. Statistical analysis was performed using Student's *t*-test. The criterion for statistical significance was *p* < 0.05.

### Clinical tissue samples

Paraffin embedded slides were procured from Emory University Hospital and Northside Hospital with information on clinical outcomes. The Emory Institutional Review Board (IRB) and Northside Hospital approval was obtained for all aspects of the study. Twenty fresh-frozen tissue samples for immunoblotting were procured from Meenakshi V. Gupta, West Georgia Hospital. All the tissue samples used were archived and de-identified, thus no patient consent was required.

### Immunofluorescence, imaging and scoring

Tissue slides were de-paraffinized by baking at 67°C for 2 h followed by 3 xylene washes and rehydrated in a series of ethanol baths (100%, 95%, 70% and 50%). Antigen retrieval was performed by citrate buffer (pH 6.0) in a pressure cooker for 3min. Primary antibody incubation was done overnight against γ-tubulin (1:1000) at 4°C. The samples were incubated with secondary antibody (Alexa-488 anti-mouse) at 37°C for 2 h. Samples were washed 3x with PBS and then mounted with Prolong-Gold antifade reagent that contained DAPI (Invitrogen). Tissue samples were imaged using the Zeiss LSM 700 Confocal microscope (Oberkochen, Germany) and images were processed with Zen software (Oberkochen, Germany). Percentage cells with centrosomal abnormality were quantitated from 10 randomly selected fields per sample (500 cells counted for each sample).

### Immunohistochemistry and scoring

Samples were processed in the same way till antigen retrieval as mentioned above in Immunofluorescence section. Tissues were then immunostained for vimentin, Plk4 and Aurora-A. To perform enzymatic antibody detection, Universal LSAB + kit/HRP (DAKO, CA, USA) was used. A relative intensity score was represented as 0 = none, 1 = low, 2 = moderate, or 3 = high and frequency score was depicted as the percentage of cell nuclei or cytoplasm demonstrating vimentin positivity (i.e. a score of 1, 2, or 3). The product of intensity and frequency was measured as weighted index (WI) for both the nucleus and cytoplasm.

### Three-dimensional volume measurement

10–15 fields of view within the stained tissue were imaged via laser scanning confocal microscopy (Zeiss, Oberkochen, Germany) and for each field, optical sections of 0.5 um thickness each are acquired. For each field of view, the optical sections are stacked to produce a “maximum intensity projection image”. The raw confocal images are then opened in a 3-D volume rendering software (Zeiss, Oberkochen, Germany) for determining the volumes of each centrosome present in each microscopic field. The volume range for a normal centrosome is determined by analyzing the volumes of at least 500 centrosomes from normal tissue of 3 normal breast tissues. The smallest and the largest value for centrosomal volume provide the normal centrosome volume range for that tissue.

### Cell culture

MCF-7, MDA-MB-231 and MDA-MB-468 cells were purchased from ATCC. MCF-7cells were grown in MEM, MDA-MB-231 and MDA-MB-468 were grown in DMEM supplemented with 10% Hyclone Fetal Bovine serum and 1% penicillin/streptomycin and MCF-10-A were grown in MGEM (Lonza). All cell lines were maintained in 5% CO_2_ atmosphere at 37°C.

### Cellular protein preparation, Immunoblot analysis, Immunofluorescence and antibodies

Protein lysates were prepared from ~70% confluence cells and frozen tissue samples (sonicated). Briefly, PAGE was used to resolve the proteins and transferred on to PVDF membrane (Millipore). Pierce ECL detection kit (Thermo Scientific) was used to visualize the immune-reactive bands corresponding to respective primary antibodies. *β*-actin was used as loading control. For immunofluorescence staining, cells were grown on glass coverslips and fixed with ice cold methanol for 10 min. Blocking was done by incubating with 2% bovine serum albumin/PBS.0.05% Triton X-100 at 37°C for 1 h. Primary Antibodies against γ-tubulin, α-tubulin were incubated with coverslips for 1 h at 37°C at the dilution 1:2000. The cells were washed with 2% bovine serum albumin/PBS for 10 min at room temperature before incubating with a 1:2000 dilution of Alexa 488- or 555-conjugated secondary antibodies Invitrogen (Carlsbad, CA, USA). Antibodies against γ- tubulin, α-tubulin and vimentin were from Sigma (St. Louis, MO, USA), Aurora-A, Plk4 and *β*-actin were from cell signaling, Centrin-2 and Cyclin E from Santacruz and Pericentrin-2 from Abcam. Horseradish peroxidase-conjugated secondary antibodies were from Santa Cruz Biotechnology (Santa Cruz, CA, USA).

### Boyden chamber assay

MCF-10A cells overexpressing inducible Plk4 or truncated version of Plk4 (1-608) were collected after 24 h transfection and resuspended in media at 5 × 10^4^ cells/ml density. Transmigration assay was carried out in a Boyden chamber system. Cells that had migrated to the bottom surface of the filter were fixed with 70% methanol, stained with crystal violet, and counted under a microscope in 10 randomly selected fields at 20X magnification (Zeiss Axioplan).

### Time-lapse imaging

Stably transfected centrin-GFP MDA-MB-231 cells were plated at 60% confluence on Matek cell culture plates. Cells were then imaged using time-lapse microscopy at 40X magnification on PerkinElmer Ultra View ERS spinning disc microscope (Waltham, MA, USA). GFP images were captured at multiple points every 10 min for 18 h. Captured images from each experiment were analyzed using Volocity software (Improvision Coverty, UK).

### Statistical analysis

Statistical analysis was performed using student t-test and ANOVA, where the criteria for statistical significance was *p* < 0.05. For ideal thresholds, the FINDCUT macro developed by Jayawant N. Mandrekar et al. from Mayo Clinic (http://www2.sas.com/proceedings/sugi28/261-28.pdf) was used, which identifies the optimal cut off point for a continuous variable that predicts time to event outcomes, in our case CAI and %CA.

## SUPPLEMENTARY FIGURE AND TABLE


