# Supplementary material for: Rampant centrosome amplification underlies more aggressive disease course of triple negative breast cancers
Source: Oncotarget. 2015 Mar 19;6(12):10487–97. doi: 10.18632/oncotarget.3402 (PMC4496369; doi:10.18632/oncotarget.3402)
Supplement: Supplementary file 1 [file oncotarget-06-10487-s001.pdf]

SUPPLEMENTARY FIGURE AND TABLE

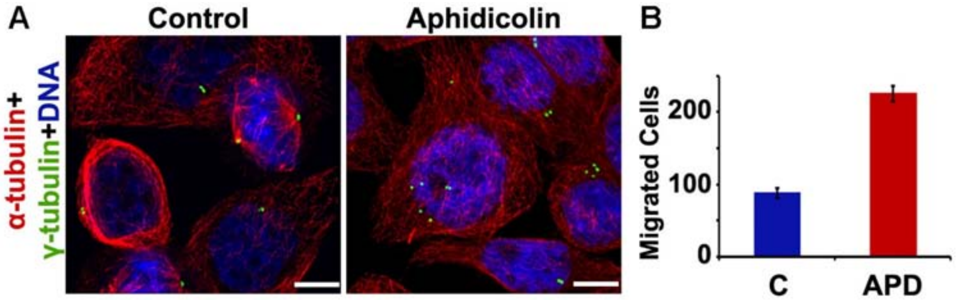

Supplementary Figure S1: Cells with amplified centrosomes have higher invasion capacity. (A) Immunofluorescence micrographs showing MDA-MB-231 cells treated with DMSO (left) and MDA-MB-231 cells treated with 5ug/ml aphidicolin (right), stained for γ-tubulin (green), α-tubulin (red) and DAPI. **(B)** Bar graph showing the number of migrated cells in Boyden chamber assay performed with treated and untreated MDA-MB-231 cells.

Supplementary Table S1. List of the GSE ID's for TCGA and GEO database used in analysis

**Non-TNBC GEO Series ID Grade**	**TNBC GEO Series ID Grade**
GSE7390	GSE2990 GSE6532 GSE19615 GSE18864 GSE19615
**Non-TNBC GEO Series ID Stage**	**TNBC GEO Series ID Stage**
GSE2109 GSE37751 GSE39004	GSE2109 GSE37751 GSE39004
**Survival plot data**	
GSE37751 GSE39004	
